# Network analysis of psychological capital: mapping interconnected dynamics in teacher wellbeing and professional commitment

**DOI:** 10.3389/fpsyg.2026.1784108

**Published:** 2026-06-09

**Authors:** Yanran Zhang, Zahari Ishak, Bicheng Diao

**Affiliations:** 1 Department of Culture and Tourism, Jining Polytechnic, Jining, Shandong Province, China; 2 Faculty of Social Sciences and Liberal Arts UCSI University, Kuala Lumpur, Malaysia; 3 School of International Education, Lianyungang Normal University, Lianyungang, Jiangsu Province, China

**Keywords:** bridge centrality, network analysis, professional commitment, psychological capital, teacher wellbeing

## Abstract

**Introduction:**

Junior high school teachers increasingly face occupational stressors linked to heightened anxiety and attrition, underscoring the urgency to identify psychological resources that sustain wellbeing and professional commitment. While psychological capital (PsyCap), comprising self-efficacy, hope, resilience, and optimism, is recognized as a buffer against burnout, prior studies often treat it monolithically, neglecting sub_dimensional dynamics and contextual moderators.

**Methods:**

A total of 1,271 junior high school teachers participated in this study. They completed a 26_item PsyCap scale, the 15_item PERMA Questionnaire (measuring positive emotion, engagement, relationships, meaning, and accomplishment), and a 16_item Professional Commitment (PC) Questionnaire. Network analysis was employed to examine the interrelationships among PsyCap sub_dimensions, wellbeing components, and professional commitment.

**Results:**

Network analysis revealed that positive emotion demonstrated the highest strength centrality, suggesting its central position within the network structure. Self_efficacy and resilience also played important roles as key psychological resources linking psychological capital and teacher wellbeing. Hope exhibited high bridge centrality, connecting goal_oriented pathways to career commitment, whereas optimism showed limited bridging roles, potentially reflecting cultural inclinations toward pragmatic hope over abstract optimism. Node strength demonstrated moderate_to_high stability (CS_coefficient = 0.749), but betweenness centrality declined in subsampling analyses (50% subsampling: *r* = 0.45), revealing context_dependent fragility.

**Discussion:**

These findings advance a sub_dimension_specific framework, emphasizing that dynamic interactions, rather than aggregate PsyCap scores, drive educators' vocational resilience. Methodologically, network analysis outperformed traditional latent variable models in capturing non_linear dynamics. Culturally tailored interventions prioritizing hope and resilience, rather than generalized optimism, are recommended to optimize teacher support systems. This study highlights the critical role of context_sensitive, network_informed approaches in occupational health research and policy design.

## 1 Introduction

Middle school teachers play an important but challenging role in education worldwide. They support students during a key growth period but face many challenges. The middle school stage is a critical period for students‘ physical and mental growth. Teachers also must handle the complex emotional and psychological problems of students (Miao et al., 2023; Wang et al., 2024). After the implementation of the “Double Reduction policy” in China, the workload of primary and secondary school teachers has increased. 72% of the teachers work for more than 10 h every day. Influenced by traditional Confucian education, society has high expectations for teachers (Qian et al., 2024). Due to the uneven distribution of educational resources, parents in underdeveloped areas place all their hopes for their children's future on teachers (Xu and Zhu, 2022; Bai and Wang, 2022). Therefore, teachers must undertake teaching tasks and face pressure from parents and society. These challenges have eroded teachers' professional identity, contributing to rising attrition rates. Recent studies show serious concerns. About 42% of new teachers have high anxiety levels, and more than 15% leave their jobs within 5 years in stressful regions (Wang, 2023; UNESCO, 2023; Li J. et al., 2023). This problem harms education and costs a lot of money. In the U.S., replacing teachers costs about $2.2 billion each year (Sutcher et al., 2019). To solve this issue, researchers focus on psychological resources that help teachers stay in their jobs. One key resource is psychological capital (PsyCap). It includes self-confidence, hope, resilience, and optimism. Studies show that PsyCap can reduce teacher burnout (Luthans et al., 2007). However, some experts say looking at PsyCap as one single idea is a problem. Each part of PsyCap may affect teachers differently. Their impact may also depend on different work conditions (Jeong and Kim, 2022; Xu and Yang, 2024). The conceptualization of psychological capital (PsyCap) as a positive state-like psychological resource that plays a dual role in enhancing career resilience and buffering burnout has gained empirical support as a result (Luthans et al., 2004). Across occupations, moderate to strong associations have been found between global psychological resilience and reduced job satisfaction, task performance, and intention to leave (Avey et al., 2011; Muylaert et al., 2023). However, there has always been a limitation in this literature: conflating the PsyCap sub-dimensions of self-efficacy, hope, resilience, and optimism in a composite score, thereby obscuring their distinct functional roles and synergistic interactions (Cui, 2022). Emerging evidence from cross-cultural research challenges this monolithic approach. For example, resilience is more strongly associated with career commitment in collectivist educational settings, whereas self-efficacy dominates in individualistic settings, suggesting a cultural moderating effect of sub-dimension salience (Cheng and Zhao, 2023; Wang, 2024).

Wellbeing is an important idea in occupational health research. It includes emotional stability, personal growth, and life purpose (Diener et al., 2003). In education, teachers' wellbeing affects teaching quality and student success. However, it is often harmed by long-term stress (Gan and Cheng, 2021). Professional commitment means how emotionally connected a person is to their job. It helps turn wellbeing into long-term job engagement (Meyer et al., 1993). Even though these ideas are connected, most studies look at them separately. Many studies use simple research methods, such as cross-sectional surveys and linear models, which may not fully explain complex relationships (Borsboom and Cramer, 2013). For example, resilience helps reduce burnout, while optimism is linked to proactive coping. However, treating psychological capital (PsyCap) as one single factor may hide these differences (Li M. et al., 2023). New research suggests a systems approach to studying psychological processes. This approach looks at how different factors interact instead of studying them separately (Lewis, 2011). In complex systems, behavior comes from relationships between parts, not just the parts themselves. The system network model applies this thinking by viewing psychological traits as connected elements rather than separate categories (Burke et al., 2015).

Adopting a network-based framework represents a pivotal advancement in elucidating the interconnected dynamics among psychological capital (PsyCap), wellbeing, and professional commitment. Conventional studies predominantly employ reductionist models to examine PsyCap's four subdimensions (self-efficacy, hope, resilience, and optimism) as isolated components. However, emerging evidence suggests these subdimensions operate through synergistic interactions rather than linear additive effects. For instance, resilience may not exert a direct influence on career commitment; instead, it could amplify hope, which subsequently enhances self-efficacy, thereby collectively fortifying persistence amid adversities. Such cascading mediation pathways remain obscured in traditional regression or latent variable frameworks. Similarly, while wellbeing is frequently conceptualized as a distal outcome in PsyCap research, network theory posits its role as a proximal mediator hub—simultaneously assimilating inputs from PsyCap dimensions and channeling motivational resources toward behavioral commitment.

The system network model provides methodological robustness to disentangle these multilevel interdependencies: intra-level associations, cross-level bridging effect, and bidirectional feedback loops can be empirically mapped without presupposing hierarchical causal orders (Sekhar, 2022). Network analysis represents a paradigm shift in psychological research, moving beyond reductionist “variable-centered” frameworks to model psychosocial ecosystems as dynamic systems of interacting elements (Borsboom, 2017). Unlike latent variable approaches that assume homogeneity, this method quantifies constructs through interconnected nodes, revealing emergent properties such as central hubs and bridge variables that drive systemic behavior (Robinaugh et al., 2016). By simultaneously incorporating multiple interacting factors, network models uncover complex patterns often obscured in traditional analyses (Epskamp et al., 2018). For instance, centrality metrics (strength, betweenness) provide actionable insights into intervention targets: strengthening a bridge node like resilience, which connects psychological capital (PsyCap) to wellbeing, may propagate cascading benefits across the network. Empirical applications have advanced occupational health research, including mapping burnout trajectories (Gürbüz et al., 2023) and leadership dynamics (Kalisch et al., 2019). Despite these advances, the sub-dimensional architecture of PsyCap networks remains poorly characterized, limiting tailored interventions for high-stress professions.

This study investigates the network architecture and dynamic interrelationships among psychological capital (PsyCap) subdimensions—self-efficacy, hope, resilience, and optimism—in shaping educator wellbeing and professional commitment. Unlike traditional latent variable models that assume homogeneity, network analysis quantifies constructs as interconnected nodes, enabling the identification of central hubs and bridge effects (Borsboom, 2017). Building on the network theory of positive psychology, we utilize Gaussian graphical modeling (GGM) and directed acyclic graphs (DAGs) to delineate core nodes and bridge connections within the integrated PsyCap-wellbeing-commitment system. The findings aim to reconceptualize PsyCap's role in sustaining educational ecosystems by identifying actionable pathways for psychological resource optimization. Specifically, the study highlights how context-dependent interactions between PsyCap subdimensions influence occupational resilience and institutional engagement. These insights are expected to advance a precision-focused framework for cultivating adaptive psychological resources in high-stress educational settings, with implications for policy design and intervention strategies tailored to mitigate educator attrition. However, existing research has largely failed to explain how different PsyCap subdimensions dynamically interact to sustain teachers' wellbeing and professional commitment under high occupational stress, due to the dominance of reductionist and variable-centered analytical frameworks.

Unlike traditional approaches that treat psychological capital as a single latent construct, this study adopts a network analysis perspective to investigate the interactions among its subdimensions and related psychological outcomes.

Specifically, this study seeks to answer the following research questions:

RQ1: What are the structural relationships among the dimensions of psychological capital, teacher wellbeing, and professional commitment within the psychological network of junior high school teachers?
RQ2: Which components of psychological capital occupy central positions in the network?
RQ3: Which dimensions function as bridge nodes connecting psychological capital, wellbeing, and professional commitment?

Accordingly, the objectives of this study to examine the network structure among psychological capital, wellbeing, and professional commitment, to identify the central psychological capital components within the network, and to determine the bridge nodes that link these psychological constructs. By identifying key nodes within the network, this study aims to provide a more nuanced understanding of how psychological resources support teachers' occupational wellbeing and professional commitment. By explicitly mapping these relationships, the study seeks to inform targeted interventions that optimize teachers' psychological resources, enhance wellbeing, and strengthen professional commitment.

## 2 Material and methods

### 2.1 Participants and procedure

This study employed a stratified sampling strategy, collecting data through the Chinese platform “Questionnaire Star”. To ensure data quality, all teachers participating in the survey have more than 1 year of teaching experience, and the survey is anonymous. If participants feel any discomfort during the study, they can withdraw at any time. All participants signed informed consent forms before the survey and could opt out at any time to safeguard their informed and voluntary involvement. A total of 1,271 middle school teachers were included in the formal analysis. The institutional ethics committee approved this study under the reference code [IEC-2024-FOSSLA-0039].

### 2.2 Measurements

#### 2.2.1 Psychological capital (PsyCap)

Psychological capital (PsyCap) was assessed using the 26-item Psychological Capital Questionnaire (PCQ-26) developed by (Zhang et al. 2010) in the Chinese educational context. Five reverse-scored items (Item 8: “I often feel powerless in the classroom”) were included to mitigate response bias. Participants rated statements on a 5-point Likert scale (1 = strongly disagree to 5 = strongly agree). In this study, the Cronbach's α coefficient was 0.935 for Psycap.

#### 2.2.2 Wellbeing (PERMA)

Wellbeing was assessed using the validated Chinese version of the PERMA-Profiler (Butler and Kern, 2016), a multidimensional instrument grounded in Seligman's (2011) PERMA theory of wellbeing. This framework encompasses five domains: Positive Emotion, Engagement, Relationships, Meaning, and Accomplishment. Sample items include:Meaning: “Do you feel your life has purpose and significance?” Accomplishment: “How often do you feel progress toward your personal goals?” Responses were collected on a 5-point Likert scale ranging from 1 (never) to 5 (always), with elevated scores indicating higher levels of wellbeing. The PERMA-Profiler demonstrated good internal consistency in the current sample, with a Cronbach coefficient of 0.817 for the overall scale.

#### 2.2.3 Professional commitment (PC)

Professional commitment was measured via the 16-item Teacher Professional Commitment Scale (Long and Li, 2002), which integrates Meyer et al.'s (1993) tripartite model into the Chinese educational context. Three reverse-scored items (“I regret choosing this profession”) were included to enhance response accuracy. Participants rated items on a 5-point Likert scale (1 = strongly disagree to 5 = strongly agree), with higher scores indicating stronger commitment. In this study, the Cronbach's α coefficient was 0.917 for PC.

### 2.3 Statistical analysis

#### 2.3.1 Network estimation

The network structure among psychological capital (PsyCap) subdimensions, wellbeing, and professional commitment was estimated using the EBICglasso algorithm (Extended Bayesian Information Criterion Graphical LASSO) via the qgraph package in R (Epskamp et al., 2018). This method combines LASSO regularization with an EBIC tuning parameter (γ = 0.5) to optimize network sparsity while minimizing false-positive edges, particularly suited for high-dimensional psychological data (Foygel and Drton, 2010). Partial correlations between variables were computed, controlling for all other nodes in the network, with edge weights thresholded at |0.15| to retain clinically meaningful associations (Friedman et al., 2008). The algorithm iteratively shrinks trivial edges to zero, producing a parsimonious network that balances interpretability and accuracy. Regularization parameters were selected through 10-fold cross-validation to ensure robustness. Network stability was further verified via 1,000-case bootstrapping, with centrality indices (strength, betweenness) retained only if correlation stability coefficients exceeded 0.50 (Epskamp and Fried, 2018).

#### 2.3.2 Estimation of centrality and bridge centrality

After estimating the network, we measured node centrality to find the most important psychological factors. We calculated three centrality measures: degree centrality, betweenness centrality, and closeness centrality. Degree centrality counts how many direct links a node has. A node with a high degree of centrality connects to many other nodes and has a strong influence (Freeman, 1979; Santos et al., 2021). Betweenness centrality shows how often a node is on the shortest path between other nodes. A high betweenness centrality means the node acts as a bridge or mediator (Freeman, 1977; Borgatti and Halgin, 2021). Closeness centrality measures how near a node is to all other nodes in the network. Nodes with high closeness centrality can quickly spread information (Borgatti, 2005).

It is important to note that betweenness centrality is less stable than other centrality indicators in psychological networks, particularly in smaller samples or subsample analyses (Epskamp et al., 2018; Bringmann et al., 2019). Therefore, while we report betweenness centrality for descriptive purposes, our interpretation of node importance primarily relies on strength centrality, which has demonstrated greater robustness in simulation studies.

Bridge centrality finds nodes that connect different groups in the network. The strength of these bridges is measured by their contribution to cross-group connections (Marsman et al., 2018; Bringmann et al., 2023).

#### 2.3.3 Network accuracy and stability estimation

To test the reliability of the network, we used bootstrap analysis (1,000 times) and case-dropping with the bootnet package (Epskamp, 2020). Bootstrap analysis created many random samples to estimate 95% confidence intervals for edge weights and centrality measures (strength, betweenness). This helped check the accuracy of our results (Efron and Hastie, 2021). Case-dropping tested how stable the network structure was. We removed one case at a time and rebuilt the network. This showed which edges and nodes remained strong despite changes in the sample (Williams and Rast, 2020). We also calculated correlation stability (CS) coefficients. We kept measures with CS ≥ 0.50, meaning they had moderate stability (Christensen et al., 2020). These methods jointly ensured the network's replicability, with unstable edges (CIs overlapping zero) flagged for cautious interpretation.

#### 2.3.4 Principal component analysis and euclidean distance modeling

To further examine the structural coherence of the psychological capital (PsyCap) subdimensions, wellbeing, and career commitment constructs, we conducted complementary exploratory analyses using SPSS.

First, we performed Principal Component Analysis (PCA) with Varimax rotation to explore the underlying dimensionality of the measured constructs. It is important to note that PCA operates within the latent variable paradigm, which differs from the network analysis paradigm. Therefore, this analysis is presented not as a structural validation of the network, but as an auxiliary exploration to provide convergent evidence regarding construct dimensionality. The PCA results offer a complementary perspective on how the items cluster together, which can be compared with the network structure identified in the main analysis.

Second, we employed Euclidean distance modeling within SPSS to quantify the topological dissimilarity between subgroup-specific networks. This geometric approach provides a spatial interpretation of structural consistency across different groups, complementing the network analysis by assessing whether network structures remain stable across subsamples (Borsboom et al., 2021). Unlike PCA, Euclidean distance modeling aligns with the network paradigm as it operates on the relational patterns between nodes rather than on latent factor extraction.

Together, these complementary analyses provide a multi-perspective examination of the data, enhancing our understanding of the structural properties of the PsyCap-wellbeing-commitment system without conflating latent variable and network approaches.

## 3 Results

### 3.1 Descriptive statistics

**Figure 1:**
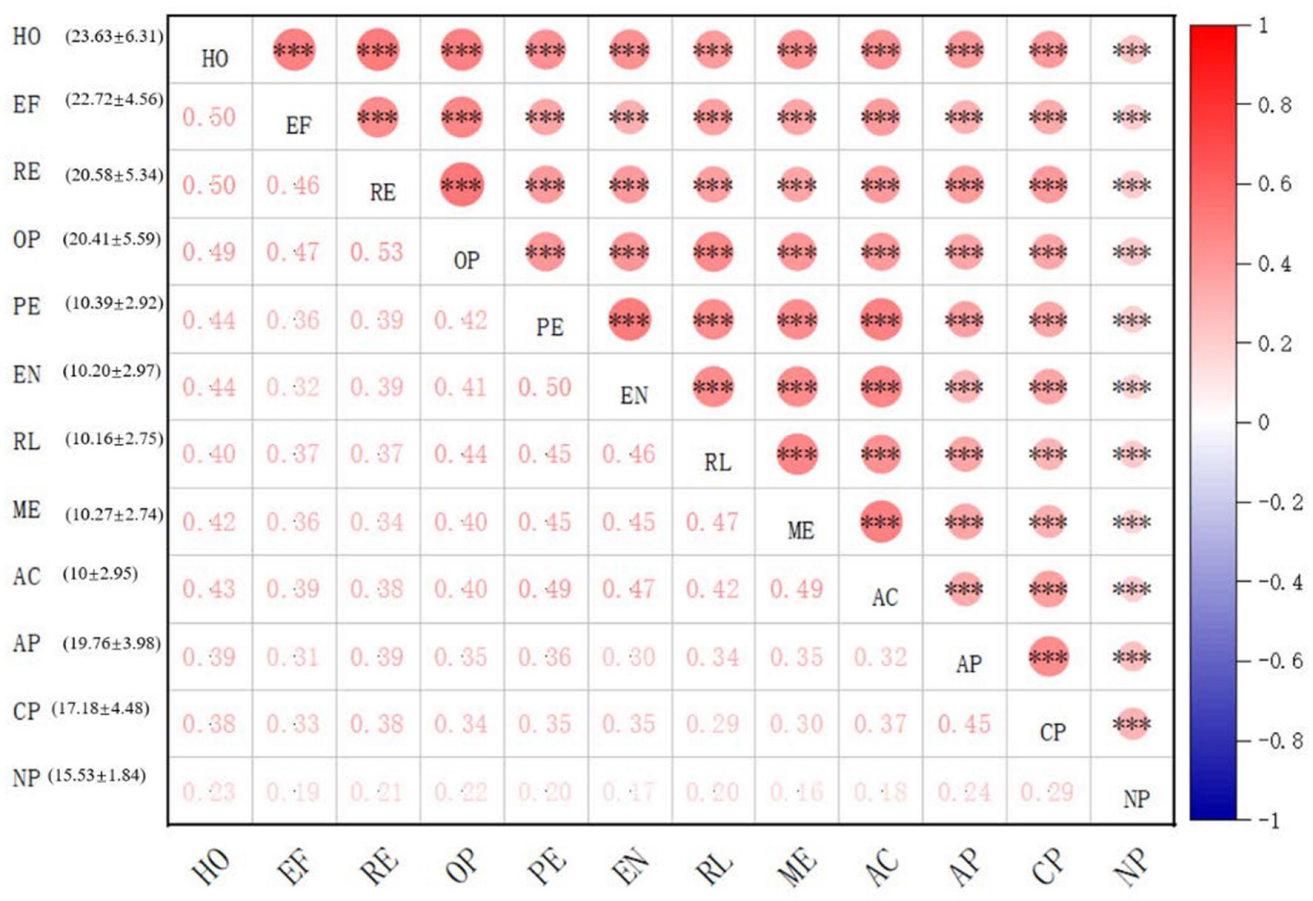
Descriptive statistical results and correlation matrix between variables. Statistical significance was set at three levels: ^***^p < 0.001.

Figure 1 presents the descriptive statistical results of the scale and the correlation matrix among the variables. The lower left corner of the matrix shows the correlation coefficients between the variables, while the upper right corner visually displays the relationships among the variables. The depth of the color and the shape of the circles indicate the strength of the correlation (where deeper colors and larger circles correspond to stronger correlations). An asterisk (^*^) indicates that the corresponding correlation coefficient has a significance level of *p* < 0.05. The results show that the correlation coefficients between most variables range from 0.30 to 0.50 and are statistically significant (*p* < 0.05), indicating a moderate positive correlation among the internal variables of the scale.

### 3.2 Network structure

**Figure 2:**
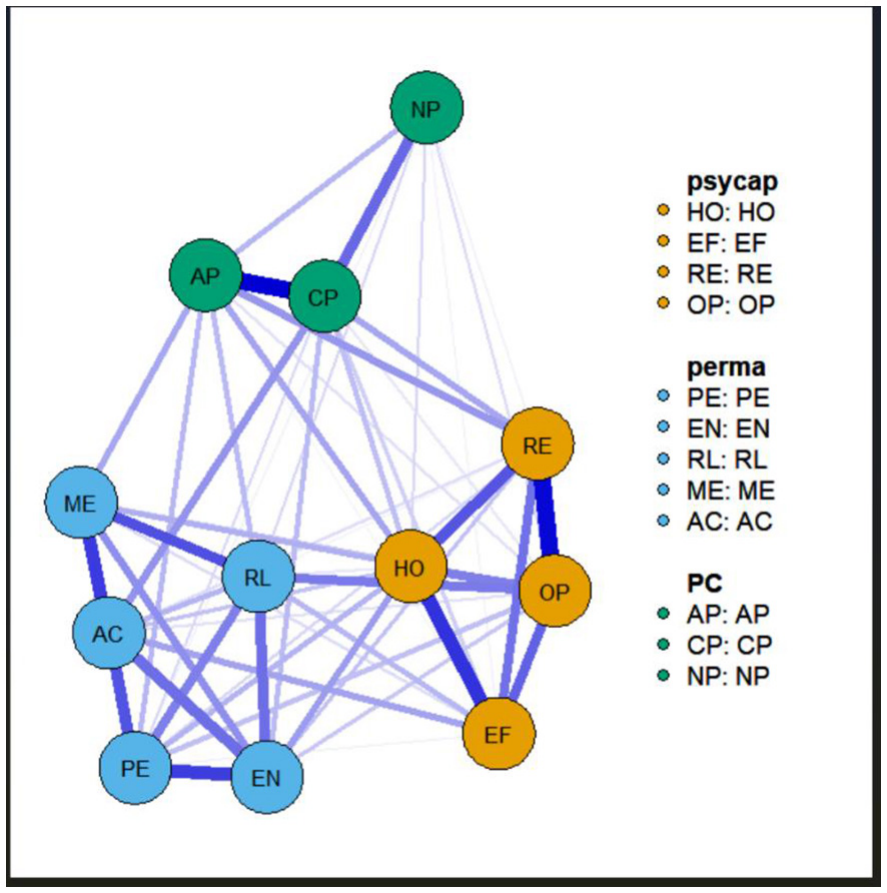
Network of psycap, wellbeing, and PC nodes in teachers.

Figure 2 illustrates the network of psychological capital, wellbeing, and professional commitment among junior high school teachers. In the psychological capital (PsyCap) and wellbeing (PERMA) network, node HO (“Hope”) demonstrated the strongest direct connection to node PE (“Positive Emotions”). This was followed by the link between nodes RE (“Resilience”) and PE (“Positive Emotions”). In the PERMA wellbeing network, node PE (“Positive Emotions”) showed the most significant direct correlation with node EN (“Engagement”), which was followed by the link between nodes EN (“Engagement”) and ME (“Meaning”). Within the professional commitment (PC) group, node CP (“Continuous Commitment”) demonstrated the strongest direct connection to node AC (“Accomplishments”). This was followed by a strong connection between nodes AP (“Affect Commitment”) and RL (“Relationships”). The inclusion of node NP (“Normative Commitment”) suggests a complex interaction with other wellbeing factors, particularly its moderate connection to ME (“Meaning”).

### 3.3 Centrality and bridge centrality

**Figure 3:**
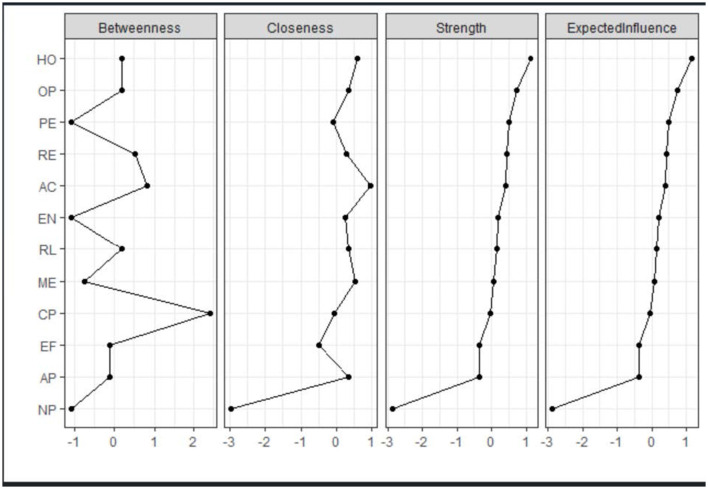
Node strength centrality within the estimated network.

To more intuitively compare the variables in the network Status, Figure 3 illustrates the centrality and bridge strength of nodes within the network of junior high school teachers. Of note, node HO (“Hope”) demonstrated the highest betweenness centrality, followed by nodes OP (“Optimism”) and PE (“Positive Emotions”). For closeness centrality, HO (“Hope”) also ranked highest. In terms of strength centrality, PE (“Positive Emotions”) demonstrated the highest strength, followed by EN (“Engagement”), indicating their strong and numerous intra-network connections. For expected influence, PE (“Positive Emotions”) was the most influential node, with EN (“Engagement”) ranking second. In terms of bridge expected influence (1-step), HO (“Hope”) was identified as the strongest bridge node, followed by EF (“Efficacy”) and RE (“Resilience”), demonstrating their pivotal role in bridging distinct network subgroups. In contrast, NP (“Normative Commitment”) consistently ranked lowest across all centrality measures. These findings highlight HO (Hope), PE (Positive Emotions), and EN (Engagement) as central hubs maintaining network cohesion, while underscoring NP's (Normative Commitment) negligible role in network dynamics, as further visualized in Supplementary Figure 1.

### 3.4 Network accuracy and stability

**Figure 4:**
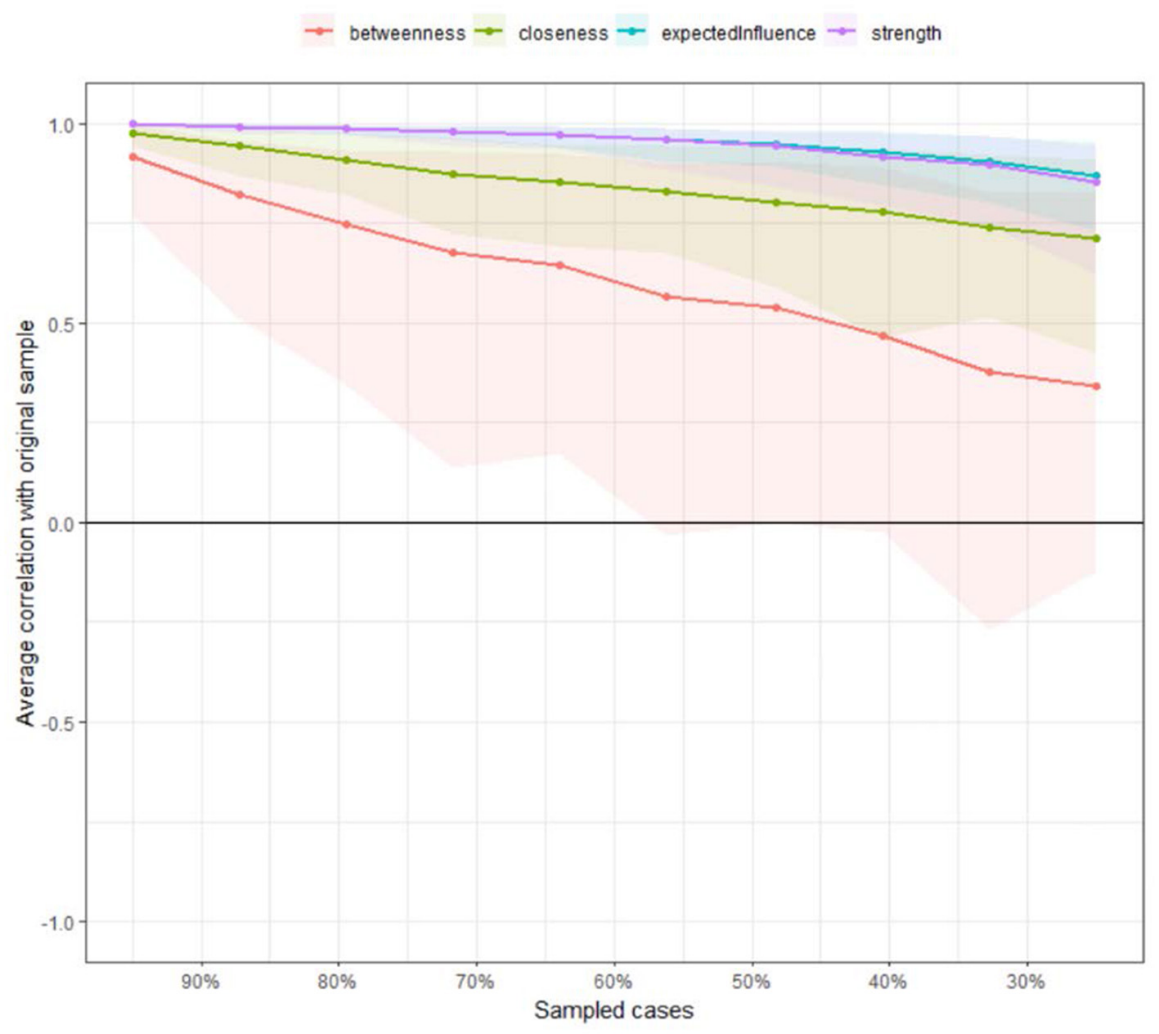
The stability of centrality indices by using case dropping subset bootstrap method.

In the cross-sectional networks, edge weight bootstrapping (Supplementary Figure 2) demonstrated moderate accuracy in estimating the networks. The CS-coefficient for strength centrality reached 0.749, indicating moderate-to-high stability (Epskamp et al., 2018), as shown in Figure 4. The overall network stability, assessed through multiple CS coefficients (Supplementary Figure 3), demonstrated high robustness, with all centrality metrics exceeding the recommended threshold of 0.5.

The assessment of edge stability revealed moderate consistency within the estimated networks. Bootstrap analysis) demonstrated substantial overlap in 95% of the edge weight confidence intervals (CIs). The non-parametric bootstrapped difference test for strength demonstrated that the nodes HO (“Hope”) and OP (“Optimistic”) had the highest strength compared to other symptoms (Supplementary Figure 4).

### 3.5 Principal component analysis

**Figure 5:**
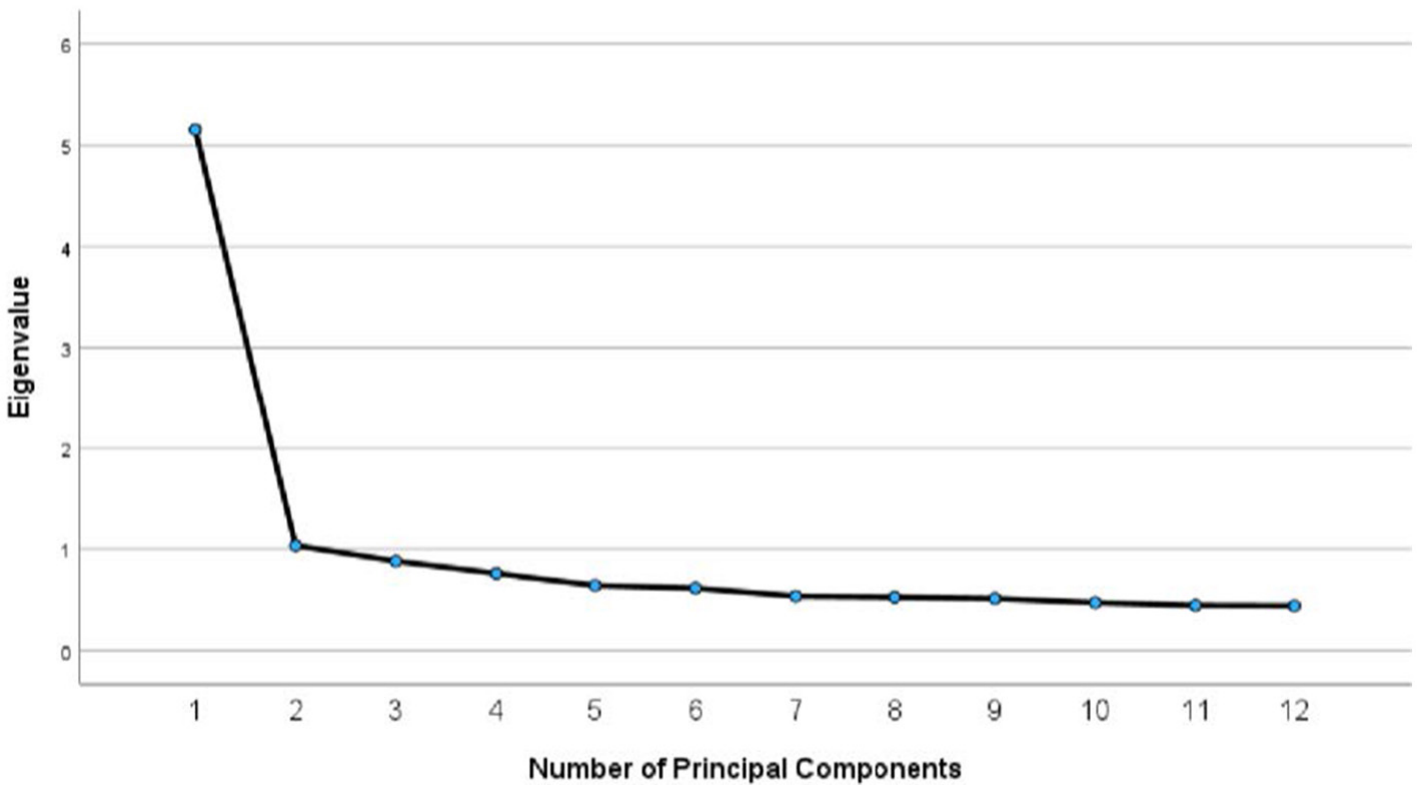
Scree plot.

To verify the reliability of the network structure obtained from the network analysis, this study further uses the principal component analysis for verification. The test results of sampling adequacy of 12 variables are KMO = 0.931 and Bartlett's of sphericity ≈ 2,665.307, which is suitable for principal component analysis. According to the 12 variables in Figure 5, two principal components with eigenvalue >1 are extracted, and the cumulative contribution rate reaches 51.570%, which is also reflected in the network diagram as two stable clusters (PsyCap + PERMA) and PC, which are the same as the two stable clusters in the network structure.

**Figure 6:**
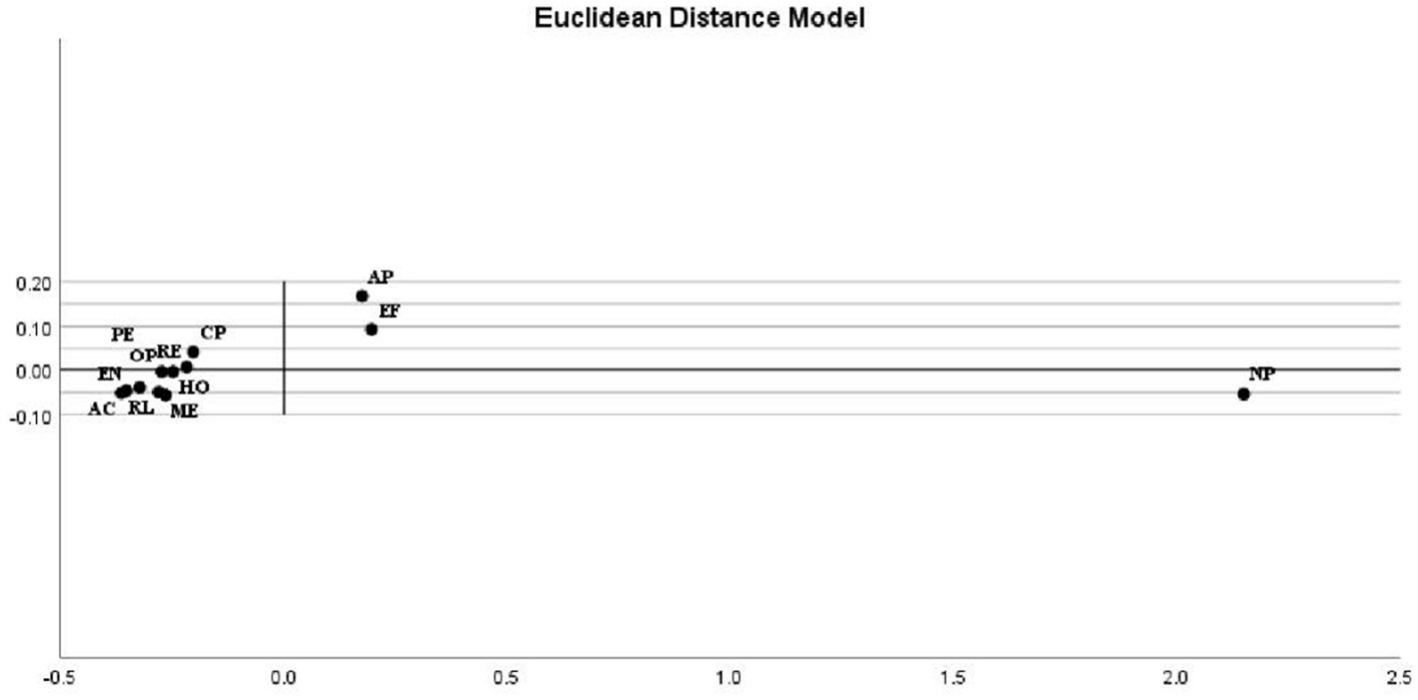
Euclidean distance model.

To further verify the reliability of the network analysis, this study employed the Euclidean Distance Model (see Figure 6) as a supplementary analysis of the relationships between variables. The results indicate that in the Euclidean distance plot, variables such as AP and EF are positioned far from the center, while NP is noticeably isolated from other variables. This pattern aligns with the centrality indices (Closeness, Betweenness) in the network analysis. It suggests that NP may play a more peripheral role in the network structure, whereas AP and EF exhibit higher connectivity. Furthermore, based on the Strength and Expected Influence indices, the structural characteristics of the network closely align with the clustering trends reflected in the Euclidean Distance Model, further supporting the validity of the network analysis.

## 4 Discussion

Based on ecosystem network theory, this study included 12 psychological factors, used network analysis to explore the relationship between the factors, and identified key influencing factors combined with the centrality index. From the whole network, it can be seen that there are clusters with close connections between two nodes in the study. The same result was obtained by further verification of principal component analysis. Our findings reveal a tightly integrated network structure, with key nodes (Hope, Positive Emotions, Engagement) acting as central hubs, while normative commitment exhibited minimal influence.

The prominence of Hope (HO) as the node with the highest betweenness and closeness centrality aligns with its theorized role as a bridging construct in PsyCap frameworks (Luthans et al., 2007). Hope's capacity to connect disparate network subgroups (linking Resilience to Positive Emotions) resonates with findings that identified hope as a mediator between adversity and adaptive outcomes in educators (Li M. et al., 2023). Similarly, the dominance of Positive Emotions (PE) in strength centrality. This finding indicates that positive emotions function as a central activating mechanism within the psychological network. And expected influence underscores their function as amplifiers of wellbeing, consistent with (Sriwidharmanely et al. 2022) updated broaden-and-build theory, which posits that positive emotions enhance cognitive flexibility and social resource accumulation.

Notably, the strong PE–EN (Engagement) linkage mirrors recent work, who demonstrated that educators' positive affective states directly fuel sustained engagement in high-stress environments. Each cluster encompasses factors at different levels, including emotions, personality, and beliefs. The connections between the two clusters are established through associations among these multi-level factors, further confirming that career commitment is a phenomenon involving a multi-layered system that necessitates an integrated perspective for comprehensive understanding.

Focus on the peripheral nodes. The consistently low centrality of Normative Commitment (NP) across all metrics challenges conventional assumptions about its role in teacher retention. While prior studies framed normative commitment as a moral anchor, our network analysis reveals its limited functional integration with wellbeing or PsyCap nodes (Houle et al., 2022). This discordance may reflect shifting sociocultural dynamics: in collectivist contexts like China (where this study was conducted), normative obligations are increasingly supplanted by affective and calculative commitments due to rising workloads and bureaucratic pressures (Rodríguez-Fernández et al., 2024).

This finding aligns with cross-cultural research by (Wang et al. 2024), who reported declining normative commitment among Asian educators post-COVID-19. However, NP's moderate connection to Meaning (ME) suggests residual alignment with purpose-driven motivations.

This study found that the bridge centrality of OP in Chinese teachers was significantly lower than the results reported in Western studies (Scheier et al., 2021), reflecting the profound influence of cultural values on the expression of psychological capital (PsyCap). This phenomenon can be explained from the perspectives of cultural psychology and educational practice. In a collectivist cultural context, Chinese teachers may emphasize “pragmatic hope,” focusing on specific goals and collective responsibilities, rather than the “abstract optimism” highlighted in Western PsyCap frameworks.

Pragmatic hope is typically linked to specific goals and collective responsibilities, whereas abstract optimism focuses more on generalized positive expectations for the future. As a result, OP plays a weaker cross-domain connecting role in the psychological network of Chinese teachers, functioning primarily within specific educational contexts rather than as a generalized psychological resource. Similarly, the high-intensity assessment environment in Chinese schools might influence how optimism functions within professional networks. These interpretations remain speculative hypotheses and should be empirically tested in future cross-cultural studies. Such research could clarify whether cultural scripts and educational practices systematically shape the expression and network integration of PsyCap components in teaching contexts. Research suggests that in high-pressure environments, teachers are more likely to rely on immediate problem-solving strategies and stress-buffering mechanisms rather than long-term optimistic beliefs (Yuan et al., 2025). This adaptive strategy may weaken OP's role as a central hub within the PsyCap network. Cultural factors may influence the expression of PsyCap in Chinese teachers. One possible explanation is the so-called “modesty norm” in traditional Chinese culture, which could lead teachers to express optimism more implicitly rather than verbally. For instance, teachers might convey confidence through concrete actions, such as enhancing lesson planning, rather than through explicit statements of positive expectations. If this interpretation holds, such implicit expression could partially explain the relatively lower cross-domain connectivity of optimism (OP) observed in our network, although further empirical investigation is needed to confirm this hypothesis.

The moderate-to-high stability of node strength and edge weights (95% CI overlap) suggests robustness in core network features, yet declining centrality correlations at lower subsampling ratios underscore the fragility of betweenness metrics. These results echo methodological critiques by (Borsboom 2017), who cautioned against overinterpreting betweenness centrality in sparse or small-sample networks. The instability of weaker edges (EF–NP, RL–AP) aligns with (Chen et al. 2022), who attributed such variability to context-dependent interactions in social systems. To enhance reproducibility, future studies could adopt multilevel network modeling (MLNM) to disentangle within–and between-school effects. MLNM can distinguish between individual-level and organizational-level interactions and reduce confusion variation.

Our findings suggest targeted interventions focusing on key nodes. Hope-centric training, incorporating goal setting and pathway-thinking exercises, could enhance network cohesion due to HO's bridging role. Integrating positive psychology exercises with engagement strategies may further improve wellbeing and professional commitment. Additionally, administrators should prioritize affective commitment (AP) and accomplishment (AC), given their stronger network integration compared to normative commitment. These recommendations align with the International Positive Education Network's (2023) call for systemic, strengths-based approaches in educator support.

This study uses the method of network analysis to explore 12 psychological factors that affect professional commitment and obtains the association model that is difficult to get in the traditional analysis method, which provides a novel perspective for understanding the complex phenomenon of professional commitment. However, there are some shortcomings in the current research. The stability of the results of network analysis depends on the sample size. Due to the special group of subjects, the stability of some indicators in this study is not ideal, and the sample size needs to be expanded in future studies. In this study, the relative importance of factors is measured by using indicators to get the ranking. The relative importance and its difference can be further investigated in future studies. Heterogeneity testing was used to make the results more rigorous. Future research can also be based on the demographic characteristics of the teacher group, Gender construction, and analysis of more detailed networks to explore their characteristics to provide empirical support for the designation of more targeted interventions.

While traditional regression approaches offer valuable insights into the predictive relationships among variables, they often assume linearity and independence among predictors. In contrast, network analysis allows for a holistic examination of the mutual associations and interdependencies between components of psychological capital, wellbeing, and professional commitment.

For example, in our network, the node “Hope” not only showed the strongest centrality but also acted as a bridge between psychological capital and wellbeing domains. Traditional regression would identify “Hope” as a significant predictor only if its effect size exceeded others; However, it would not reveal its mediating or linking role across variable clusters. Similarly, the marginal role of “Normative Commitment” observed in our network may go unnoticed in regression models but becomes evident through its low centrality and peripheral position. Therefore, network analysis reveals relational structures and dynamic interactions among psychological variables that are not easily captured by traditional linear models. This justifies the use of a more complex analytic method, given its ability to generate novel insights that inform theory and practice in educational psychology.

## 5 Limitations and future research directions

This study has several limitations that should be acknowledged. First, the cross-sectional design limits the ability to draw causal inferences regarding the relationships among psychological capital, wellbeing, and professional commitment. Future research could employ longitudinal approaches, such as cross-lagged panel models or experience sampling methods, to examine how these relationships evolve. Second, all variables were measured using self-report questionnaires, which may introduce common method bias. Although Harman's single-factor test indicated that common method variance was not a major concern, future studies could strengthen validity by incorporating multiple data sources, such as peer evaluations or observational measures.

Third, the findings should be interpreted within the cultural context of China, which may influence the expression and interaction of psychological capital components. Cross-cultural comparative studies are therefore needed to examine the generalizability of the network structure identified in this study.Finally, the sample consisted only of junior high school teachers, which may limit the generalizability of the results to other educational levels or institutional contexts. Future research could examine whether similar network patterns emerge across different teacher populations and educational settings.

## 6 Conclusion

This study contributes to the literature by conceptualizing teachers' psychological functioning as an interconnected system, integrating psychological capital (PsyCap), PERMA wellbeing, and professional commitment within a network framework. The identification of hope and positive emotions as central nodes highlights key leverage points that sustain the resilience of the system, while the peripheral role of normative commitment challenges traditional assumptions about obligation-based retention. From a theoretical perspective, the findings move beyond linear models by demonstrating that these constructs are structurally interdependent rather than independent predictors. This system-oriented perspective provides a more nuanced understanding of how psychological resources and professional attitudes interact within educational contexts. Methodologically, this study underscores the innovative value of network analysis in capturing complex interrelationships among psychological variables. By identifying central and peripheral nodes, the network approach offers insights that may be overlooked in traditional latent variable models, thereby enriching analytical approaches in educational research. Looking ahead, future research should further explore the dynamic and contextual nature of these psychological networks, particularly how they evolve across time and settings, and how targeted interventions on central nodes may generate broader systemic effects.

Overall, this study provides a network-based perspective that advances both theoretical understanding and practical strategies for promoting teacher wellbeing and sustainable educational systems.

## Data Availability

The original contributions presented in the study are included in the article/supplementary material, further inquiries can be directed to the corresponding author.

## References

[B1] Avey J. B. Luthans F. Jensen S. M. (2011). Psychological capital: a positive resource for combating employee stress and turnover. Hum. Resour. Manage. 48, 677–693. doi: 10.1002/hrm.20294

[B2] Bai L. Wang Y. X. (2022). Intercultural teacher–student relationships: a qualitative study of students on 2 + 2 tertiary joint programs. Aust. Educ. Res. 49, 407–423. doi: 10.1007/s13384-021-00435-x

[B3] Borgatti S. P. (2005). Centrality and network flow. Soc. Networks 27, 55–71. doi: 10.1016/j.socnet.2004.11.008

[B4] Borgatti S. P. Halgin D. S. (2021). Analyzing Social Networks Edn., 2nd Ed. Thousand Oaks, CA: SAGE Publications.

[B5] Borsboom D. (2017). A network theory of mental disorders. World Psychiatry 16, 5–13. doi: 10.1002/wps.20375 28127906 PMC5269502

[B6] Borsboom D. Cramer A. O. J. (2013). Network analysis: an integrative approach to the structure of psychopathology. Annu. Rev. Clin. Psychol. 9, 91–121. doi: 10.1146/annurev-clinpsy-050212-185608 23537483

[B7] Borsboom D. Deserno M. K. Rhemtulla M. Epskamp S. Fried E. I. McNally R. J. . (2021). Network analysis of multivariate data in psychological science. Nat. Rev. Methods Primers 1:58. doi: 10.1038/s43586-021-00055-w

[B8] Bringmann L. F. Elmer T. Epskamp S. Krause R. W. Schoch D. Wichers M. . (2019). What do centrality measures measure in psychological networks? J. Abnorm. Psychol. 128:892. doi: 10.1037/abn0000446 31318245

[B9] Bringmann L. F. Helmich M. Eronen M. Voelkle M. (2023). Complex systems approaches to psychopathology. Oxford textbook of psychopathology 4, 103–122. doi: 10.1093/med-psych/9780197542521.003.0005

[B10] Burke J. G. Lich K. H. Neal J. W. Meissner H. I. Yonas M. Mabry P. L. (2015). Enhancing dissemination and implementation research using systems science methods. Int. J. Behav. Med. 22, 283–291. doi: 10.1007/s12529-014-9417-3 24852184 PMC4363012

[B11] Butler J. Kern M. L. (2016). The PERMA-profiler: a brief multidimensional measure of flourishing. Int. J. Wellbeing 6, 1–48. doi: 10.5502/ijw.v6i3.526

[B12] Chen J. Cheng H. Zhao D. Zhou F. Chen Y. (2022). A quantitative study on the impact of working environment on the wellbeing of teachers in China's private colleges. Sci. Rep. 12:10042, doi: 10.1038/s41598-022-07246-9 35233001 PMC8888672

[B13] Cheng C. Zhao J. (2023). The impact of professional learning communities on pre-service teachers' professional commitment. Front. Psychol. 14:1153016. doi: 10.3389/fpsyg.2023.1153016 37448713 PMC10336210

[B14] Christensen A. P. Garrido L. E. Guerra-Peña K. Golino H. (2020). Comparing community detection algorithms in psychological data: a monte carlo *simulation*. Behav Res Methods 56, 1485–1505. doi: 10.31234/osf.io/hz89e 37326769

[B15] Cui L. (2022). The role of teacher–student relationships in predicting teachers' occupational wellbeing, emotional exhaustion, and enthusiasm. Front. Psychol. 13:896813. doi: 10.3389/fpsyg.2022.896813 35664194 PMC9162152

[B16] Diener E. Oishi S. Lucas R. E. (2003). Personality, culture, and subjective wellbeing: emotional and cognitive evaluations of life. Annu. Rev. Psychol. 54, 403–425. doi: 10.1146/annurev.psych.54.101601.145056 12172000

[B17] Efron B. Hastie T. (2021). Computer age statistical inference, student edition: algorithms, evidence, and data science (Vol. 6). Cambridge: Cambridge University Press.

[B18] Epskamp S. (2020). Psychometric network models from time-series and panel data. Psychometrika 85, 206–231. doi: 10.1007/s11336-020-09697-3 32162233 PMC7186258

[B19] Epskamp S. Borsboom D. Fried E. I. (2018). Estimating psychological networks and their accuracy: a tutorial paper. Behav. Res. Methods 50, 195–212. doi: 10.3758/s13428-017-0862-1 28342071 PMC5809547

[B20] Epskamp S. Fried E. I. (2018). A tutorial on regularized partial correlation networks. Psychol. Methods 23, 617–634. doi: 10.1037/met0000167 29595293

[B21] Foygel R. Drton M. (2010). Extended bayesian information criteria for Gaussian graphical models. Adv. Neural Inf. Process. Syst. 23, 604–612.

[B22] Freeman L. C. (1977). A set of measures of centrality based on betweenness. Sociometry 40, 35–41. doi: 10.2307/3033543

[B23] Freeman L. C. (1979). Centrality in social networks: conceptual clarification. Soc. Networks 1, 215–239. doi: 10.1016/0378-8733(78)90021-7

[B24] Friedman J. Hastie T. Tibshirani R. (2008). Sparse inverse covariance estimation with the graphical lasso. Biostatistics 9, 432–441. doi: 10.1093/biostatistics/kxm045 18079126 PMC3019769

[B25] Gan Y. Cheng L. (2021). Psychological capital and career commitment among Chinese urban preschool teachers: the mediating and moderating effects of subjective wellbeing. Front. Psychol. 12:509107. doi: 10.3389/fpsyg.2021.509107 34366945 PMC8339257

[B26] Gürbüz S. Bakker A. B. Demerouti E. Brouwers E. P. M. (2023). Sustainable employability and work engagement: a three-wave study. Front. Psychol. 14:1188728. doi: 10.3389/fpsyg.2023.1188728 37397284 PMC10313196

[B27] Houle S. A. Morin A. J. Fernet C. (2022). Longitudinal trajectories of affective commitment to the occupation among school principals: a person-centered perspective. J. Vocat. Behav. 137:103758. doi: 10.1016/j.jvb.2022.103758

[B28] International Positive Education Network (2023). A Strengths-Based Framework for Positive Education: Guidelines for Implementation. Dordrecht: Springer.

[B29] Jeong S. A. Kim J. (2022). Factors influencing nurses' intention to care for patients with COVID-19: focusing on positive psychological capital and nursing professionalism. PLoS ONE 17:e0262786. doi: 10.1371/journal.pone.0262786 35045117 PMC8769348

[B30] Kalisch R. Cramer A. O. J. Binder H. Fritz J. Leertouwer I. Lunansky G. . (2019). Deconstructing and reconstructing resilience: a dynamic network approach. Perspect. Psychol. Sci. 14, 765–777. doi: 10.1177/1745691619855637 31365841

[B31] Lewis T. G. (2011). Network Science: Theory and Applications. Hoboken, NJ: John Wiley & Sons.

[B32] Li J. Huang C. Yang Y. Liu J. Lin X. Pan J. (2023). How nursing students' risk perception affected their professional commitment during the COVID-19 pandemic: the mediating effects of negative emotions and moderating effects of psychological capital. Hum. Soc. Sci. Commun. 10:195. doi: 10.1057/s41599-023-01719-6 37192948 PMC10156579

[B33] Li M. Fan W. Zhang L. F. (2023). Career adaptability and career choice satisfaction: roles of career self-efficacy and socioeconomic status. Career Dev. Q. 71, 300–314. doi: 10.1002/cdq.12334

[B34] Long L. Li X. (2002). Study on professional commitment of primary and secondary school teachers. Educ. Res. Exp. 4:6.

[B35] Luthans F. Avolio B. J. Avey J. B. Norman S. M. (2007). Positive psychological capital: measurement and relationship with performance and satisfaction. Pers. Psychol. 60, 541–572. doi: 10.1111/j.1744-6570.2007.00083.x

[B36] Luthans F. Luthans K. W. Luthans B. C. (2004). Positive psychological capital: Beyond human and social capital. Bus. Horiz. 47, 45–50. doi: 10.1016/j.bushor.2003.11.007

[B37] Marsman M. Borsboom D. Kruis J. Epskamp S. van Bork R. V. Waldorp L. J. . (2018). An introduction to network psychometrics: relating ising network models to item response theory models. Multivariate Behav. Res. 53, 15–35. doi: 10.1080/00273171.2017.1379379 29111774

[B38] Meyer J. P. Allen N. J. Smith C. A. (1993). Commitment to organizations and occupations: extension and test of a three-component conceptualization. J. Appl. Psychol. 78, 538–551. doi: 10.1037/0021-9010.78.4.538

[B39] Miao D. Zhu M. Zhou Z. Zhang N. (2023). How school closures affected learning and the physical and mental health of Chinese university students during the COVID-19 pandemic?. Build. Environ. 242:110582. doi: 10.1016/j.buildenv.2023.110582

[B40] Muylaert J. Decramer A. Audenaert M. (2023). Organizational support and emotional commitment among teachers: the role of communication protocols. Public Manag. Rev. 25, 2402–2427. doi: 10.1080/14719037.2023.2291797

[B41] Qian H. Walker A. Chen S. (2024). The ‘Double-Reduction'education policy in China: three prevailing narratives. J. Educ. Policy 39, 602–621. doi: 10.1080/02680939.2023.2222381

[B42] Robinaugh D. J. Millner A. J. McNally R. J. (2016). Identifying highly influential nodes in the complicated grief network. J. Abnorm. Psychol. 125, 747–757. doi: 10.1037/abn0000181 27505622 PMC5060093

[B43] Rodríguez-Fernández M. Herrera J. de las Heras-Rosas C. Ciruela-Lorenzo A. M. (2024). Practical implications of the organizational commitment model in healthcare: the case of nurses. J. Nurs. Manag. 2024:6455398. doi: 10.1155/2024/6455398 40224884 PMC11918622

[B44] Santos F. P. Lelkes Y. Levin S. A. (2021). Link recommendation algorithms and dynamics of polarization in online social networks. Proc. Nat. Acad. Sci. 118:e2102141118. doi: 10.1073/pnas.2102141118 34876508 PMC8685674

[B45] Scheier M. F. Swanson J. D. Barlow M. A. Greenhouse J. B. Wrosch C. Tindle H. A. (2021). Optimism vs. pessimism as predictors of physical health: a comprehensive reanalysis of *disp*ositional optimism research. Am Psychol. 76, 529–548. doi: 10.1037/amp0000666 32969677

[B46] Sekhar C. (2022). Do high-commitment work systems engage employees? Mediating role of psychological capital. Int. J. Organ. Anal. 30, 1000–1018. doi: 10.1108/IJOA-10-2020-2466

[B47] Seligman M. E. P. (2011). Flourish: A Visionary New Understanding of Happiness and Wellbeing. New York, NY: Free Press.

[B48] Sriwidharmanely S. Sumiyana S. Mustakini J. H. Nahartyo E. (2022). Encouraging positive emotions to cope with technostress's adverse effects: insights into the broaden-and-build theory. Behav. Inf. Technol. 41, 2201–2214. doi: 10.1080/0144929X.2021.1955008

[B49] Sutcher L. Darling-Hammond L. Carver-Thomas D. (2019). Understanding teacher shortages: an analysis of teacher supply and demand in the United States. Educ. Policy Anal. Arch. 27:35. doi: 10.14507/epaa.27.3696

[B50] UNESCO (2023). Global Education Monitoring Report 2023: Teachers in Crisis Contexts. Paris: UNESCOPublishing. p. 45. Available online at: https://unesdoc.unesco.org/ (Accessed July 26, 2023).

[B51] Wang X. (2023). Exploring positive teacher-student relationships: the synergy of teacher mindfulness and emotional intelligence. Front. Psychol. 14:1301786. doi: 10.3389/fpsyg.2023.1301786 38094701 PMC10716249

[B52] Wang Y. (2024). Exploring the impact of workload, organizational support, and work engagement on teachers' psychological wellbeing: a structural equation modeling approach. Front. Psychol. 14:1345740. doi: 10.3389/fpsyg.2023.1345740 38314257 PMC10834696

[B53] Wang Y. Xu W. Guan K. Zhao J. Wu P. (2024). english teachers' post-pandemic motivation in macau's higher education system. Theory Pract. Lang. Stud. 14, 1990–2001. doi: 10.17507/tpls.1407.05

[B54] Williams D. R. Rast P. (2020). Back to the basics: rethinking partial correlation network methodology. Br. J. Math. Stat. Psychol. 73, 187–212. doi: 10.1111/bmsp.12173 31206621 PMC8572131

[B55] Xu L. Zhu X. (2022). The predictive role of Chinese English as a foreign language teachers' psychological capital in their job commitment and academic optimism. Front. Psychol. 13:916433. doi: 10.3389/fpsyg.2022.916433 35923728 PMC9341325

[B56] Xu Z. Yang F. (2024). The dual-level effects of authentic leadership on teacher wellbeing: the mediating role of psychological availability. Pers. Rev. 53, 929–943. doi: 10.1108/PR-11-2021-0792

[B57] Yuan X. Mao Y. Xu X. Peng R. Tang M. Dai G. Wang B. (2025). The relationship between resilience and mental health: mobile phone dependence and its differences across levels of parent-child conflict among left-behind adolescents: a cross-sectional network analysis. BMC Public Health 25:940. doi: 10.1186/s12889-025-22105-8 40065295 PMC11892170

[B58] Zhang K. Zhang S. Dong Y. (2010). Positive psychological capital: measurement and its relationship with mental health. Stud. Psychol. Behav. 8, 58–64. Available online at: https://psybeh.tjnu.edu.cn/EN/

