# Correction: Network analysis of psychological capital: mapping interconnected dynamics in teacher wellbeing and professional commitment

**DOI:** 10.3389/fpsyg.2026.1907150

**Published:** 2026-07-07

**Authors:** Yanran Zhang, Zahari Ishak, Bicheng Diao

**Affiliations:** 1 Department of Culture and Tourism, Jining Polytechnic, Jining, Shandong Province, China; 2 Faculty of Social Sciences and Liberal Arts UCSI University, Kuala Lumpur, Malaysia; 3 School of International Education, Lianyungang Normal University, Lianyungang, Jiangsu Province, China

**Keywords:** bridge centrality, network analysis, professional commitment, psychological capital, teacher wellbeing

In the published article, affiliation “Department of Culture and Tourism, Jining Polytechnic, Jining, Shandong Province, China” was omitted for author Yanran Zhang. This affiliation has now been added for author Yanran Zhang.

Also the author Yanran Zhang was erroneously assigned to affiliation Jining Vocational and Technical College. This affiliation has now been removed for author Yanran Zhang.

The original version of this article has been updated.

