# Supplementary material for: Network analysis of psychological capital: mapping interconnected dynamics in teacher wellbeing and professional commitment
Source: Front Psychol. 2026 Jun 9;17:1784108. doi: 10.3389/fpsyg.2026.1784108 (PMC13289671; doi:10.3389/fpsyg.2026.1784108)
Supplement: Supplementary file 1 [file Image_1.pdf]

Network Analysis of Psychological Capital Subdimensions: Unraveling Dynamic Pathways to Teacher Well-being and Professional Commitment

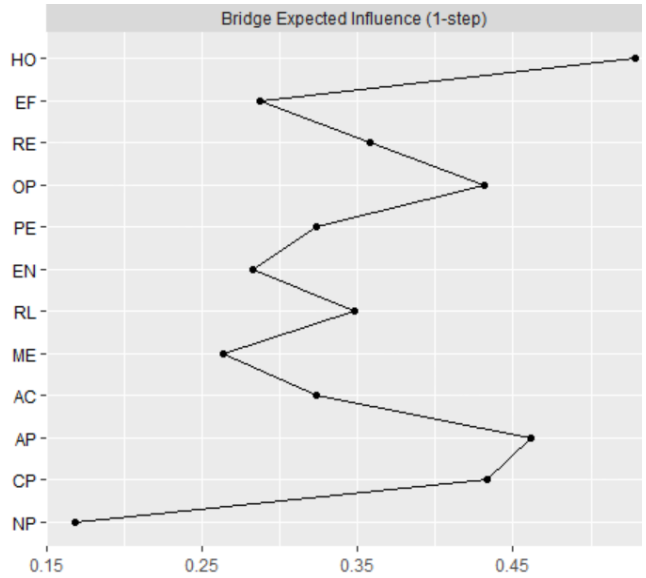

Figure S1 The bridge symptoms of PsyCap,well-being and professional commitment among teachers

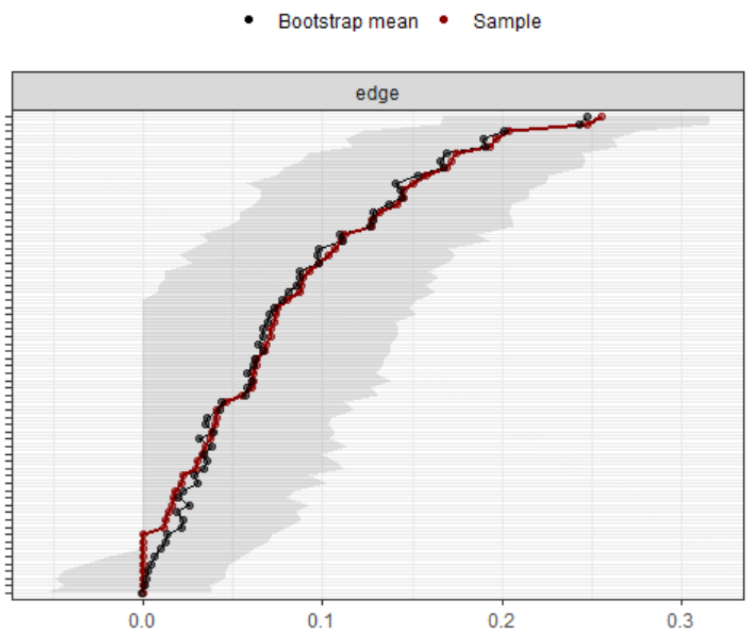

Figure S2 Bootstrapped confidence intervals of edge weights**.**

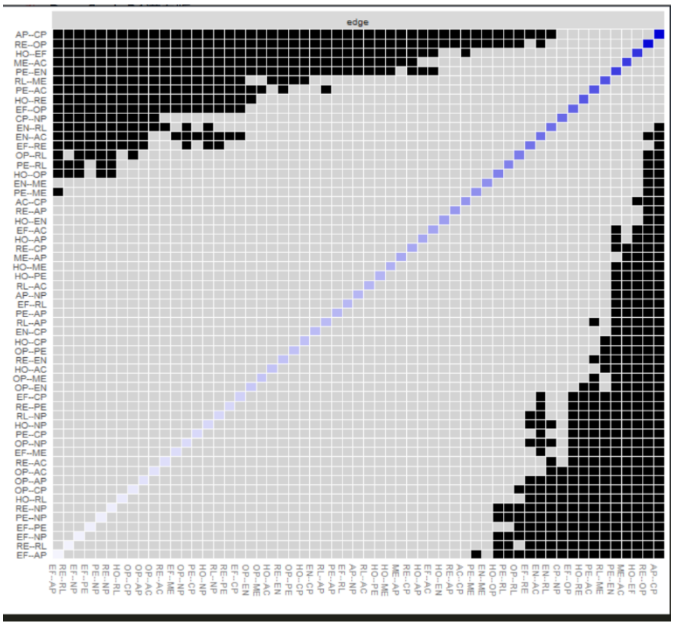

Figure S3. Estimation of edge weight difference by bootstrapped difference test.

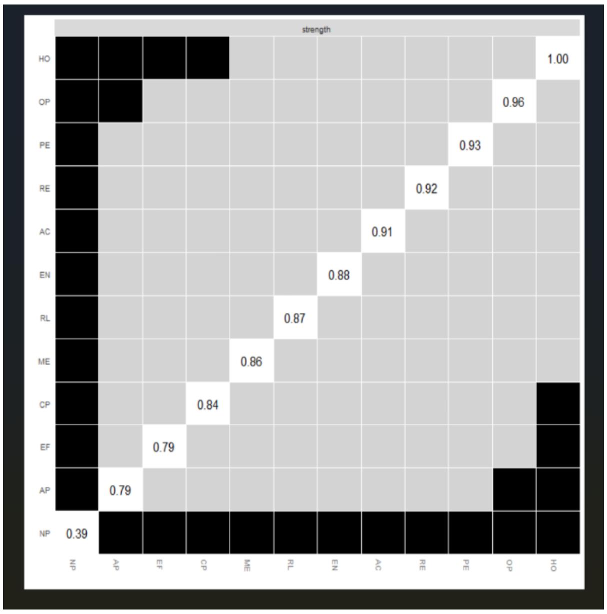

S4: Estimation of node strength difference by bootstrapped difference test.
